# Bioremediation of heavy oily sludge: a microcosms study

**DOI:** 10.1007/s10532-022-10006-1

**Published:** 2022-12-04

**Authors:** Cinthya Rondon-Afanador, Gustavo Pinilla-Meza, Francy C. Casallas-Cuervo, Camila Diaz-Vanegas, Daniela Barreto-Gomez, Carolina Benavides, Nicole Buitrago, Melissa Calvo, Camila Forero-Forero, Valentina Galvis-Ibarra, Victoria Moscoso-Urdaneta, Maria C. Perdomo-Rengifo, Laura Torres, Ziv Arbeli, Robin L. Brigmon, Fabio Roldan

**Affiliations:** 1 Facultad de Ciencias, Departamento de Biología, Unidad de Saneamiento y Biotecnología Ambiental (USBA), Pontificia Universidad Javeriana, Carrera 7 No. 43-82, Bogotá, DC Colombia; 2 Savannah River National Laboratory, Aiken, SC USA

**Keywords:** Bioaugmentation, Biodegradation, Biostimulation, Heavy oily sludge, Microcosms, Total petroleum hydrocarbons

## Abstract

Oily sludge is a residue from the petroleum industry composed of a mixture of sand, water, metals, and high content of hydrocarbons (HCs). The heavy oily sludge used in this study originated from Colombian crude oil with high density and low American Petroleum Institute (API) gravity. The residual waste from heavy oil processing was subject to thermal and centrifugal extraction, resulting in heavy oily sludge with very high density and viscosity. Biodegradation of the total petroleum hydrocarbons (TPH) was tested in microcosms using several bioremediation approaches, including: biostimulation with bulking agents and nutrients, the surfactant Tween 80, and bioaugmentation. Select HC degrading bacteria were isolated based on their ability to grow and produce clear zones on different HCs. Degradation of TPH in the microcosms was monitored gravimetrically and with gas chromatography (GC). The TPH removal in all treatments ranged between 2 and 67%, regardless of the addition of microbial consortiums, amendments, or surfactants within the tested parameters. The results of this study demonstrated that bioremediation of heavy oily sludge presents greater challenges to achieve regulatory requirements. Additional physicochemical treatments analysis to remediate this recalcitrant material may be required to achieve a desirable degradation rate.

**Supplementary Information:**

The online version contains supplementary material available at 10.1007/s10532-022-10006-1.

## Introduction

Oily sludge is the main residue generated during the extraction, transport, storage and refining of crude oil (Hu et al. 2013). Generally, these residues are composed of water (15–50%), hydrocarbons (HCs) (30–80%), solids (5–40%), and metals (Hu et al. 2013). The physicochemical composition of the sludge varies according to: (1) the type of crude oil (light or heavy), (2) the origin of the crude oil, (3) the process where the residue was generated, and (4) chemical or physical treatment used to pre-process the oily sludge (Hu et al. 2013; Aguelmous et al. 2019). The amount of oily sludge generated varies between 0.2 and 0.5% w/w of processed crude (Hu et al. 2013; Aguelmous et al. 2019). Due to the complex composition of the oily sludge, here we define ‘heavy oily sludge’ as all the solid residues from heavy oil [American Petroleum Institute (API) gravity < 20°] that has been previously processed for oil recovery.

Presence of toxic substances like benzene, polycyclic aromatic hydrocarbons (PAH), toxic byproducts, and metals in the sludge are a concern to human health and the environment (Barraza et al. 2018; Maurice et al. 2019). For this reason, several alternatives have been implemented in recent years to reduce the formation of oily sludge, recover any oil of commercial value, and treat it prior to final disposal (Hu et al. 2013; Das et al. 2018; Johnson and Affam 2019). Nevertheless, the environmentally important process of extracting energetically valuable HCs increases the viscosity and the proportion of heavier HCs in the residual oily sludge making its treatment more challenging.

Among treatment options for heavy oily sludge, physicochemical treatments removes a significant amount of the HCs present in the oily sludge but they are expensive and hazardous due to the reagents involved (Hu et al. 2013; Jasmine and Mukherji 2019; Johnson and Affam 2019). Bioremediation is an effective and sustainable alternative that has been used for the treatment of contaminated sites with HCs and oily sludge. Yet, the success of this type of treatment depends on the biodegradability and bioavailability of the contaminants and may be inhibited by the presence of toxic agents. For oily sludge, the most common strategies of bioremediation are biostimulation and bioaugmentation, which have been implemented at laboratory and field scale (Ouyang et al. 2005; Ayotamuno et al. 2010; Cerqueira et al. 2014). For oily sludge, the most widely used bioremediation methodologies are landfarming, biopiles, composting, and bioreactors (Da Silva et al. 2012; Hu et al. 2013; Aguelmous et al. 2019). However, few studies reported the use of bioremediation during the treatment of heavy oily sludge and more studies are needed in regions where this oil is produced.

Biostimulation consists of identifying and supplying biodegradation limiting factors, such as nutrients, terminal electron acceptors, co-substrates, bulking agents, bioavailability of the contaminant, or pH adjustment in order to stimulate microbial activity, and enhance the degradation of the contaminant (King et al. 1997; Hurst and Crawford 2002; Tyagi et al. 2011). The addition of nutrients, especially nitrogen and phosphorus, with organic or inorganic fertilizers has been used successfully to enhance the degradation of total petroleum hydrocarbons (TPH) (Tahhan et al. 2011; Cerqueira et al. 2014). Moreover, depending on the contaminant and microbial metabolism to be stimulated, terminal electron acceptors such as oxygen in aerobic processes or nitrate, ferric iron or sulfate in anaerobic processes can be supplied to the media (EPA 2001; Prince and Walters 2016). The use of bulking agents such as local agricultural residues in the biodegradation of oily sludge improves porosity, aeration, bioavailability, and nutrient diffusion (Wang et al. 2012). In addition, the bulking agents can supply nutrients necessary to stimulate bioremediation (Sayara et al. 2010; Gomez and Sartaj 2013) and may contribute with a high microbial load. Some bulking agents used for the biodegradation of HCs and oily sludge are wood chips (Marin et al. 2006), cotton stalks (Wang et al. 2012), sawdust (Ayotamuno et al. 2010; Beškoski et al. 2011; Ma et al. 2016), compost (Trejo-Hernández et al. 2007; Gomez and Sartaj 2013), and cane bagasse wastes (Trejo-Hernández et al. 2007). Other studies have evaluated the addition of surfactants, either synthetic or biologic, to improve the bioavailability of HCs present in the oily sludge (Milne et al. 1998; Gojgic-Cvijovic et al. 2012; Atagana 2015; Jasmine and Mukherji 2019). Finally, since the pH is an important factor for microbial metabolism and bioavailability of nutrients, it is recommended to keep pH within a range of 6–8 (Roldán-Carrillo et al. 2012; Kumar et al. 2019).

During bioaugmentation, pure strains or microbial consortia, are added to the remediation site to enhance biodegradation of more recalcitrant contaminants (e.g., oily sludge). The principle of operation is that the added microorganisms accelerate the degradation of the pollutant because they are well adapted to metabolize the contaminant (King et al. 1997; Hurst and Crawford 2002; Tyagi et al. 2011). Yet, bioaugmentation has not always been successful due to the inability of added microorganisms to thrive in the contaminated materials site (Thompson et al. 2005). Nevertheless, there are also reports where bioaugmentation was successful in the laboratory and control field conditions for improving degradation rates compared to biostimulation (Mancera-López et al. 2008; Gomez and Sartaj 2013; Guarino et al. 2017; Wu et al. 2017). Generally, for the isolation of degrading microorganisms, enrichment cultures are carried out in a minimal medium of salts supplemented with HCs as a sole source of carbon and energy (Cerqueira et al. 2011; Ma et al. 2015; Obi et al. 2016). After enrichment, the microorganisms can be selected based on growth in the presence of a specific compound (Ma et al. 2015), detection of specific genes (e.g., cathecol-2,3-dioxygenase genes) (Obi et al. 2016), metabolic capabilities, respiratory activity and biodegradation assays (Cerqueira et al. 2011).

Even though these bioremediation strategies have been widely used in the degradation of oily sludge (Wang et al. 2012; Dörr de Quadros et al. 2016; Asgari et al. 2017), few studies have evaluated the degradation of heavy oily sludge after a high temperature centrifugation, leaving the sludge with a high concentration of HCs. This study alternates different strategies (biostimulation and bioaugmentation) with a high batch number of assays to determine which is the best approach to degrade heavy oily sludge and thus be able to implement future field treatments. The objective of the presented work was to evaluate the effect of biostimulation and bioaugmentation for biodegradation of high concentrations (≥ 40,000 mg kg^−1^_dw_) of heavy oily sludge under laboratory conditions using microcosms with selected bulking agents, nutrients, and degrader microorganisms.

## Materials and methods

### Sampling and characterization of the heavy oily sludge

The heavy oily sludge batches were collected from the El Recreo treatment plant (ATP Ingenieria SAS) located on the municipality of San Carlos de Guaroa (latitude: 3.881804; longitude: − 73.361825) in Meta, Colombia. The oily sludge received at the site was subjected to high temperature centrifugation (100–110 °C) to recover commercially valuable oil, making this heavy oily sludge highly recalcitrant. Approximately 2 kg of heavy oily sludge from ten batches were collected and stored at room temperature (20 °C) until further use. The physiochemical characterization of one batch is provided below for illustrative purposes (Table 1). The complete TPH characterization for all batches can be found in supplementary material (Supplementary Table S1). Additionally, the concentrations of metals in four batches of heavy oily sludge are summarized in Supplementary Table S2.

**Table 1:** Physicochemical characteristics of heavy oily sludge

Parameter		Concentration	Standard deviation
Total petroleum hydrocarbons (TPH)^a^ by gas chromatography	mg kg^−1^_dw_	75,300	(11,300)
Total petroleum hydrocarbons (TPH)^b^ by gravimetric method		125,900	(15,800)
Short chain aliphatics C_9_–C_18_^a^		11,000	(3000)
Long chain aliphatics C_19_–C_36_^a^		26,300	(1650)
Aromatics C_11_–C_22_^a^		4500	(180)
*Aromatic compounds *			
Acenaphthene^a^		397.8	(90.0)
Fluorene^a^		283.6	(5.2)
Phenanthrene^a^		245.2	(11.0)
Fluoranthene^a^		206.2	(8.5)
Benzo (b) fluoranthene^a^		86.2	(8.0)
*Hydrocarbon fractions *	% _(dw)_		
Saturated^c^		44.8	(15.0)
Aromatics^c^		22.3	(5.4)
Resins^c^		23.7	(9.6)
Asphaltenes^c^		10.2	(3.9)
Humidity^d^		53.7	(1.5)

Standard deviation values are in parentheses

^a^ Method for the determination of extractable petroleum hydrocarbons (EPH) (MADEP 2004) (n = 3), except for fluorene and benzo (b) fluoranthene (n = 2)

^b^ Method 9071b (EPA 1998) (n = 3)

^c^ Method D6560 and D2007 (ASTM 2017, 2019a) (n = 4)

^d^ Method D2216 (ASTM 2019b) (n = 3)

### Isolation and selection of degrading microorganism for bioaugmentation studies

The HCs degrading microorganisms were isolated from several enrichment cultures using Bushnell Hass (BH) minimal salts media, containing (g L^−1^): MgSO_4_, 0.2; CaCl_2_, 0.02; KH_2_PO_4_, 1; K_2_HPO_4_, 1; HN_4_NO_3_, 1, and FeCl_2_, 0.05, with 2.5 g of heavy oily sludge as inoculum and the only energy and carbon source. Enrichment cultures were incubated at 30 °C with constant agitation at 200 rpm. An aliquot of 25 mL was transferred every 15 days to 100 mL of fresh BH medium, supplemented with 2.5 g of heavy oily sludge. Incubation was carried out under the same temperature and shaking conditions. After 60 days incubation, serial dilutions up to a factor of 10^−6^ were plated on BH agar with filter paper impregnated with sterile diesel (0.22 μm filtered) for bacterial isolation; and malt extract agar (MEA) supplemented with 200 mg of penicillin (Sigma Aldrich) (St. Louis, MO) and 200 µL of sterile diesel impregnated on filter paper for fungal isolation. The plates were incubated for 15 days at 30 °C and 20 °C, respectively. Successive passes were carried out in the same media and conditions until isolation of the isolates was achieved.

Growth (OD_600 nm_) of bacterial isolates in BH media with naphthalene (30 and 300 mg L^−1^), anthracene (50 and 250 mg L^−1^), toluene (30 and 100 mg L^−1^) and diesel (5% v/v), as a sole carbon source, was measured using the Bioscreen C (Growth curves Ab Ltda) (Helsinki, Finland) at 30 °C for 68 h.

Additional enrichment cultures were established with BH media inoculated with 10 g of heavy oily sludge and one of the following PAHs as the sole carbon source (reagent grade, Sigma Aldrich) (St. Louis, MO): phenanthrene (250 mg L^−1^), pyrene (250 mg L^−1^), or anthracene (125 mg L^−1^). These cultures were plated on BH agar with phenanthrene, anthracene or pyrene, which were added on the agar surface from an acetone stock solution (acetone was evaporated before inoculation), in accordance with the respective enrichment culture. Production of transparent halos around colonies were used as indication of PAH biodegradation.

Finally, we also evaluated in the bioaugmentation assays some strains with the ability to use HCs as the sole carbon and energy source from USBA’s laboratory culture collection. These strains (96, 102, 107, 111, 116, and 155) have been isolated from previous studies under different conditions and contaminants (Supplementary Table S3).

### Respiration assays

Respirometry is used to monitor microbial activity, based on O_2_ consumption and CO_2_ production during the HCs degradation. It also can determine if a contaminant exerts an inhibitory effect on microorganisms. In this study, this technique was used to evaluate the effect of different parameters in the biodegradation of the heavy oily sludge in a short period of time.

#### Determination of the initial concentration of heavy oily sludge for bioremediation studies

The effect of heavy oily sludge concentration on microbial activity was evaluated using respirometry. Soil and sawdust were mixed together (3:1 ratio) with heavy oily sludge for final TPH concentrations of 60,000, 100,000, 150,000 and 200,000 mg kg^−1^_dw_. The mixtures were placed in a respirometer consisting of a 500 mL (Youtility; Duran®) vessel equipped with a WTW OxiTop® head. The respirometers were incubated in the dark at 30 °C for 10 days. The O_2_ consumption (mg kg^−1^) of each treatment was determined based on the ideal gas law and the negative pressure generated inside the system (Komilis et al. 2010). The CO_2_ produced was trapped with 6 mL NaOH (1 N) (Karhu et al. 2009; Lamy et al. 2013).

Finally, different bulking agents (sawdust, rice husks, rice peat, chicken manure, and oven-dried cow manure) were evaluated using respirometry (Supplementary Table S4).

### Microcosm’s evaluation of bioremediation treatments

The effects of biostimulation and bioaugmentation on TPH degradation were evaluated in microcosms under laboratory conditions. Glass vessels (250 mL) with 5 g of a bulking agent and heavy oily sludge mix were used for the biostimulation tests. Periodically, three vessels were sacrificed (extracting the whole unit) for the determination of TPH. In contrast, 640 mL glass vessels with 120 g of bulking agent and heavy oily sludge mix were used for the bioaugmentation tests; three composite samples (1 g) were collected and homogenized for the TPH quantification at each sampling event. All treatments were performed in triplicates (n = 3), heavy oily sludge was first mixed with water at a 1:1 ratio to facilitate its homogenization with the bulking agent and maintaining moisture content between 30 and 60% throughout the experiments. Each microcosm had an individual wooden tongue depressor which was used weekly for homogenization and aeration.

To evaluate the degradation of the TPH present in the heavy oily sludge, the following studies were conducted:

#### Evaluation of different bulking agents and nutrients

Initially, four bulking agent mixtures (A1–A4) were evaluated to stimulate the microbial population (Table 2) (Supplementary Table S5). The selected bulking agents: soil, rice husks, pig manure and finely chopped residues of the *Elaeis guineensis* (African Palm) empty fruit bunch (raquis) were present as low-cost agricultural byproducts and found in the vicinity of the treatment plant. Some of these bulking materials had been previously assessed to stimulate the microbial metabolism in the presence of oily sludge (Marin et al. 2006; Ayotamuno et al. 2010). Each mixture was homogenized with heavy oily sludge to obtain a concentration of 50,000 mg TPH kg^−1^_dw_. Six sampling events at 0, 20, 40, 60, 90 and 120 days were carried out for the determination of TPH by Method 9071B (EPA 1998).

**Table 2:** Bulking agent mixtures used for the biostimulation experiments

Treatments	Bulking agent mixture
A1	Soil and raquis
A2	Soil, raquis, and pig manure
A3	Soil and rice husks
A4	Soil, rice husks, and pig manure
B1	Soil, organic fertilizer, sawdust, grass, cow manure, biosolids, and molasses
B2	Soil, organic fertilizer, sawdust, grass, cow manure, biosolids, and molasses
B3	Soil, organic fertilizer, grass, cow manure, raquis, biosolids, and molasses
B4	Soil, raquis, rice husks, molasses, and nutrients
B5	Soil, cow manure, rice husks, molasses, and nutrients
B6	Soil, sawdust, cow manure, raquis, rice husks, and nutrients
B7	Soil, organic fertilizer, rice husks, and nutrients
C1	Soil, sawdust, and nutrients
D1	Soil, rice husks, sugarcane bagasse, and nutrients

In the second study, seven bulking agent mixtures (B1–B7) were evaluated (Table 2) (Supplementary Table S6). The bulking agents used: were soil, organic fertilizer, rice husks, sawdust, raquis, cow manure and biosolids, which were obtained from an anaerobic digester treating the sludge of a primary clarifier. Each mixture was homogenized with heavy oily sludge to obtain a concentration of 100,000 mg TPH kg^−1^_dw_. Four sampling events at 0, 40, 90 and 160 days were carried out to determine TPH degradation using Method 9071B.

#### Evaluation of surfactant addition

To improve the HCs bioavailability, two concentrations (0.5 and 1.0% v/w) of surfactant Tween 80 (Merck) (Darmstadt, Germany) were evaluated. For this study, bulking agent mixtures B2, B5, and C1 were used (Table 2) (Supplementary Table S7). These mixtures were selected because B2 contained a high organic content, and B5 was an available material at the site. C1, with a low organic carbon content, was used to contrast B2. Each mixture was homogenized with heavy oily sludge to obtain a concentration of 100,000 mg TPH kg^−1^_dw_. Three sampling events at 0, 60 and 240 days were carried out for the determination of TPH by the MADEP Method (MADEP 2004).

#### Evaluation of different TPH concentrations

To compare the effect of TPH concentration at 60,000 and 100,000 mg kg^−1^_dw_ on the biodegradation of heavy oily sludge, bulking agent mixtures B2, B5, and C1 were assessed for TPH biodegradation. Three sampling events at 0, 40 and 130 days were carried out for the determination of TPH using Method 9071B.

#### Addition of bacterial consortia

During bioaugmentation studies, HC-degrading microorganisms were selected and added into the contaminated microcosms along with simultaneous biostimulation (e.g., nutrients or bulking agents) to increase degradation rates.

The effect of bioaugmentation on heavy oily sludge degradation was evaluated with the addition of bacterial consortia E1, E2, and E3 to the bulking agent C1 (Table 3) and compared with the biostimulation control (Supplementary Table S8). The nutrient ratio for all treatments was kept at a C:N:P ratio of 100:10:1. The heavy oily sludge and bulking agents were mixed to a concentration of 50,000 mg TPH kg^−1^_dw_. E1 inoculum was prepared as described previously (2.2 section), while the inoculum of the defined consortia E2 and E3 maintained under diesel pressure was prepared by cultivating the strains in 50 mL of nutrient broth at 150 rpm and 30 °C for 24 h.

For the E1 microcosm, the 35 mL inoculum was obtained from a 6-month enrichment culture with BH and heavy oily sludge as a carbon source. For E2 and E3, the 35 mL inoculum was prepared with an equal volume of each strain actively growing at end of the exponential phase. The inoculum concentration was estimated at 3 × 10^8^ CFU g^−1^ according to the McFarland standard No. 1. Three sampling events at 0, 30, and 60 days were performed to quantify TPH using MADEP Method.

#### Addition of the bacterial and fungal consortia

The second bioaugmentation experiment evaluated the use of bacterial and fungal consortia to promote the degradation of aromatic HCs with complex structures. Bulking agent mixture D1, containing sugarcane bagasse and nutrients (C:N:P 100:10:1), was used to stimulate fungal growth (Dong et al. 2013; Zafra et al. 2017) (Table 3) (Supplementary Table S9). The heavy oily sludge was homogenized with the bulking agent mixture to obtain a concentration of 40,000 mg TPH kg^−1^_dw_. F1 and F2 inoculum (10 mL) were prepared as described previously (2.4.4 section). For F3 inoculum, 15 disks (1 cm Ø) of potato dextrose agar (PDA) with actively growing fungal, were inoculated in 100 mL of PD broth. The cultures were incubated for 15 days at 25 °C and 120 rpm. The biomass was collected by centrifugation at 10,000 rpm, 10 min, and room temperature (± 22 °C).

The biomass was then washed with sterile DI water and mixed in a UV light sterilized blender. For microcosms F4 and F5, the inoculum was obtained by combining 5 mL of fungal consortium F3 and 5 mL of the enrichment culture F1 or bacterial consortium F2, respectively. Three sampling events at 0, 30, and 60 days were performed to quantify TPH using the MADEP Method.

**Table 3:** Bioaugmentation treatments with bacterial and fungal cultures

Bulking agent mixture	Treatments	Microorganisms
C1	C1	Without addition of microorganisms
	E1	Enrichment culture
	E2	Bacteria from the USBA collection (strains: 96, 102, 107, 111, 116)
	E3	Bacteria from heavy oily sludge (strains: 27, 201, 203, 400, 407)
D1	D1	Without addition of microorganisms
	F1	Enrichment culture
	F2	Bacterial consortium (strains: 96, 111, 116, 27, 305, 309, 401, 407)
	F3	Fungal consortium (strains: 36, 39, 54, 100, 110)
	F4	Mix F1 and F3
	F5	Mix F2 and F3

### TPH analysis

TPH microwave-assisted extraction of TPH (MARS 6; CEM) (Mathews, NC) was performed according to Method 3546 (EPA 2007) and adjusted to the manufacturer’s technical recommendations. A mixture (1:1) of acetone (HPLC grade) (Merck; Darmstadt, Germany) and dichloromethane (HPLC grade) (Scharlau; Barcelona, Spain) was used as the extraction solvent (30 mL). The extraction was conducted at 150 °C for 15 min and 100–300 W. The extract was filtered (GE Healthcare) (Chicago, IL) to an evaporation flask.

For the gravimetric determination of TPH, the Method 9071B (EPA 1998) was used. TPH determination by Gas Chromatography (GC) was conducted according to the MADEP Method (MADEP 2004) a GC (2014, Shimadzu) (Kyoto, Japan) coupled to a flame ionization detector (FID) and a HP-5 column (30 m × 0.25 mm × 0.25 μm) (Agilent; Santa Clara, CA) with helium as a gas carrier (1.86 mL min^−1^) was used. The conditions were: injection port temperature, 285 °C; detector temperature, 320 °C; and injection volume, 1 µL with a 20:1 split. The oven temperature was subjected to a ramp starting at 100 °C for 1 min and then increased to 160 °C at 8 °C min^−1^. Next, it was increased to 290 °C at 20 °C min^−1^, held for 13 min, and finally increased to 310 °C at 10 °C min^−1^ without hold, for a total run time of 30 min.

### Statistical analysis

Statistical analysis was performed using SPSS v. 22 (IBM Corp., Armonk, NY). One-way ANOVA and Tukey-Kramer were used to identify differences among treatments at the end of each experiment. The significance level was set at *p* < 0.05.

## Results

### Selection of microorganisms for the microcosm studies

Thirty-three bacterial strains were isolated and purified from enrichment cultures with heavy oily sludge as the sole carbon and energy source. In addition, 26 bacteria strains were selected from the laboratory culture collection based on their ability to grow and degrade crude oil as a carbon source. The degrading capacity of these strains was evaluated using Bioscreen C with different substrates. Nine strains, 13, 26, 27, 96, 102, 107, 111, 116 and 155, were selected based on their growth on naphthalene, anthracene, and diesel (Supplementary Fig. S1). The highest growth was observed in the presence of diesel, but only strains 155 and 107 were selected because of their significant growth on this substrate. The highest growth on PAH was present in 500 mg L^−1^ anthracene and 300 mg L^−1^ naphthalene, where strains 13, 26, 27, 96, 102, 111, and strains 107 and 111 were selected, respectively (Supplementary Fig. S1). The growth of the strains in 30 and 100 mg L^−1^ toluene was minimal, despite the biodegradation of this HC described in previous studies (Jiang et al. 2015; Rajamanickam et al. 2017).

Thirty-nine strains were isolated from the phenanthrene (8/39), anthracene (8/39) and pyrene (23/39) enrichment cultures. For the isolation of PAH degraders, colonies which formed clear halos were selected on solid BH media supplemented with the target PAH (Supplementary Fig. S2). Seven strains with different morphologies and the ability to degrade PAHs were selected: 305 and 309 on pyrene; 201 and 203 on phenanthrene; and 400, 401 and 407 on phenanthrene and anthracene (Supplementary Fig. S2).

Based on their growth and halo formation, another 16 HC degrader bacteria (Supplementary Table S3) were selected for the bioaugmentation studies.

Fourteen fungal strains were isolated from the enrichment cultures with heavy oily sludge. These fungi were grown on MEA and conserved in water until the bioaugmentation studies. An initial biodegradation screen on 50,000 mg kg^−1^_dw_ heavy oily sludge with bulking agent mix E1 showed that five of these strains (36, 39, 54, 100 and 110) were able to degrade HCs (data not shown). These fungi were selected for the bioaugmentation experiments.

### Respiration assays

#### Determination of the initial concentration of heavy oily sludge for the biodegradation studies

**Fig. 1:**
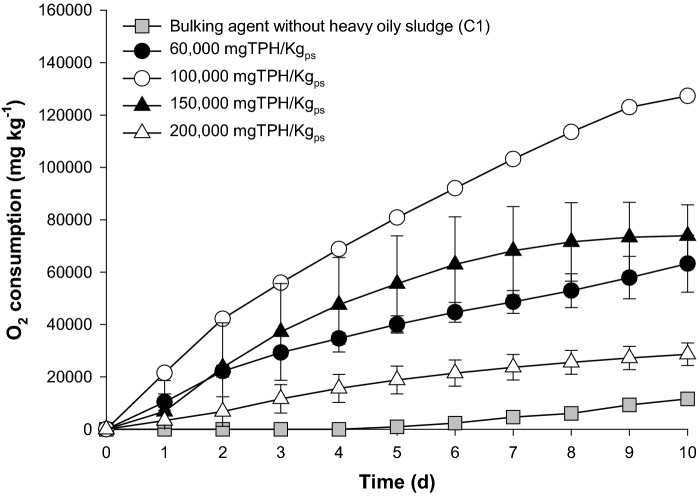
Effect of heavy oily sludge concentration on microbial activity. Error bars represent the standard deviation of three replicates

Oxygen consumption in the presence of increasing concentrations of heavy oily sludge (60,000, 100,000, 150,000 and 200,000 mg kg^−1^_dw_) was determined using soil and sawdust with a 3:1 w/w ratio as the bulking agent mixture. The highest O_2_ consumption of 120,000 ± 10,300 mg kg^−1^ at 10 days was observed with 100,000 mg TPH kg^−1^. Initial heavy oily sludge concentration stimulates microbial activity and oxygen uptake; however, higher concentrations of 150,000 and 200,000 mg kg^−1^ showed lower O_2_ consumption with a high variation, possibly due to a toxic effect on microbial activity (Fig. 1).

The bulking agent without heavy oily sludge did not show a detectable O_2_ consumption at beginning of the study (5 days) and had low consumption of 11,602 ± 2,128 mg kg^−1^ by the end of study. This indicates that the presence of heavy oily sludge was necessary to maintain an active microbial metabolism (Fig. 1).

#### Bulking agents and nutrients

**Fig. 2:**
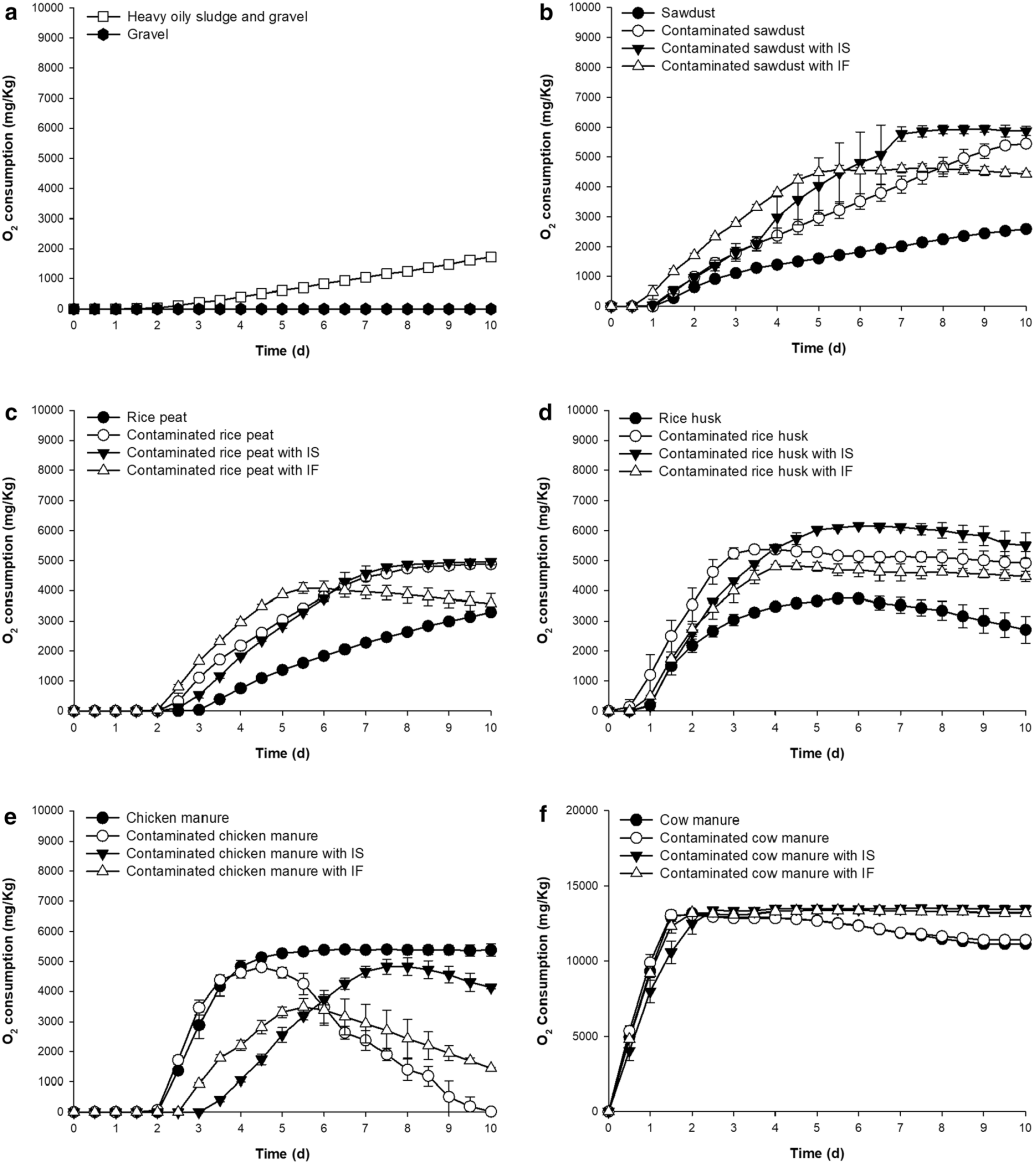
Effect of heavy oily sludge on different bulking agents’ oxygen consumption. **a** Gravel, **b** sawdust, **c** rice husks, **d** rice peat, **e** chicken manure, **f** cow manure. Error bars represent the standard deviation of three replicates. *IS *inorganic salts, *IF* inorganic fertilizer

Initially, O_2_ consumption of the bulking agents was evaluated to establish the availability of biodegradable organic substrate or, in the case of inhibition, the presence of toxic compounds. This strategy could also indicate the stimulation or inhibition for the mixture in the microcosms. While the sterile gravel did not show a detectable O_2_ consumption, the control with heavy oily sludge showed a slow O_2_ consumption, reaching a maximum value of 1,460 ± 440 mg kg^−1^ at 10 days (Fig. 2A). In addition, O_2_ consumption was determined for each bulking agent without the addition of heavy oily sludge as a control. As expected, the cow manure had the highest oxygen consumption because of the availability of organic compounds and high microbial activity. The addition of heavy oily sludge to sawdust, rice husks, and rice peat increased the O_2_ consumption, indicating an increase in microbial activity (Fig. 2B–D). On the other hand, the addition of heavy oily sludge did not increase the O_2_ consumption in the treatment with chicken manure (Fig. 2E), probably because of the toxic effect of the ammonia present in this substrate (Shen et al. 2011).

The addition of nutrients showed initial stimulation of microbial metabolism with sawdust, rice peat and rice husks; however, the maximum oxygen consumption was similar to the treatments without nutrients. Cow manure was the only bulking agent that slightly increased the oxygen consumption when nutrients were added.

### Bioremediation studies

**Fig. 3:**
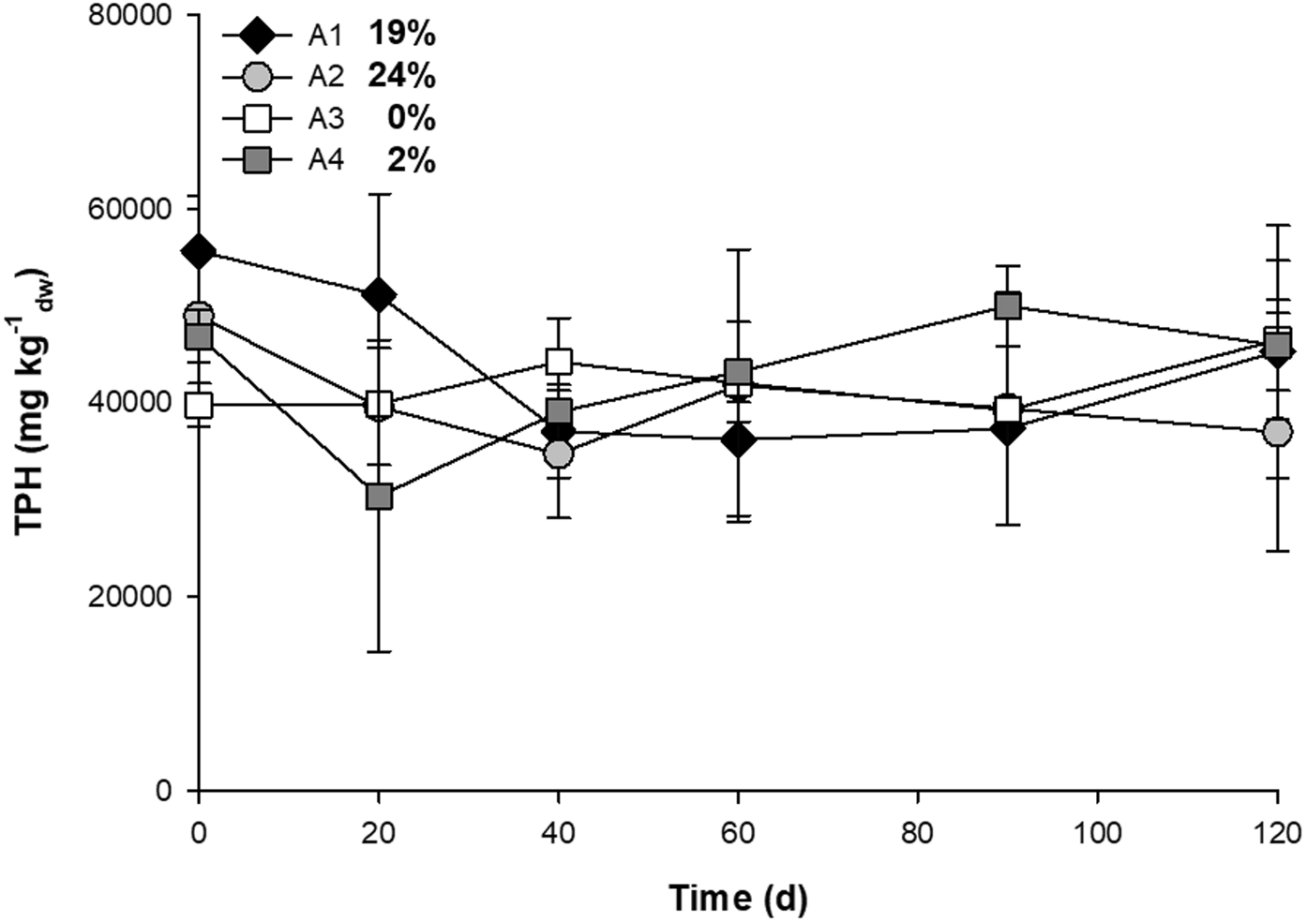
Effect of the addition of bulking agents and nutrients in the degradation of heavy oily sludge. The degradation of TPH (%) is shown next to each treatment. Error bars represent the standard deviation of three replicates. *A1 *soil and raquis, *A2* soil, raquis and pig manure, *A3* soil and rice husks, *A4* soil, rice husks and pig manure

The bulking agent mixtures initially evaluated showed no significant differences in the biodegradation of heavy oily sludge. The A2 mixture presented the highest biodegradation of 24% during the 120 days (Fig. 3), where the TPH decreased from 48,900 to 37,000 mg kg^−1^_dw_ by the end of study. The lowest biodegradation of heavy oily sludge was observed with the bulking agent mixtures with the addition of rice husks, mixtures A3 and A4. In all bulking agent mixtures, high variability in the concentration of the TPH was evidenced despite efforts in homogenization and sampling (Fig. 3).

**Fig. 4:**
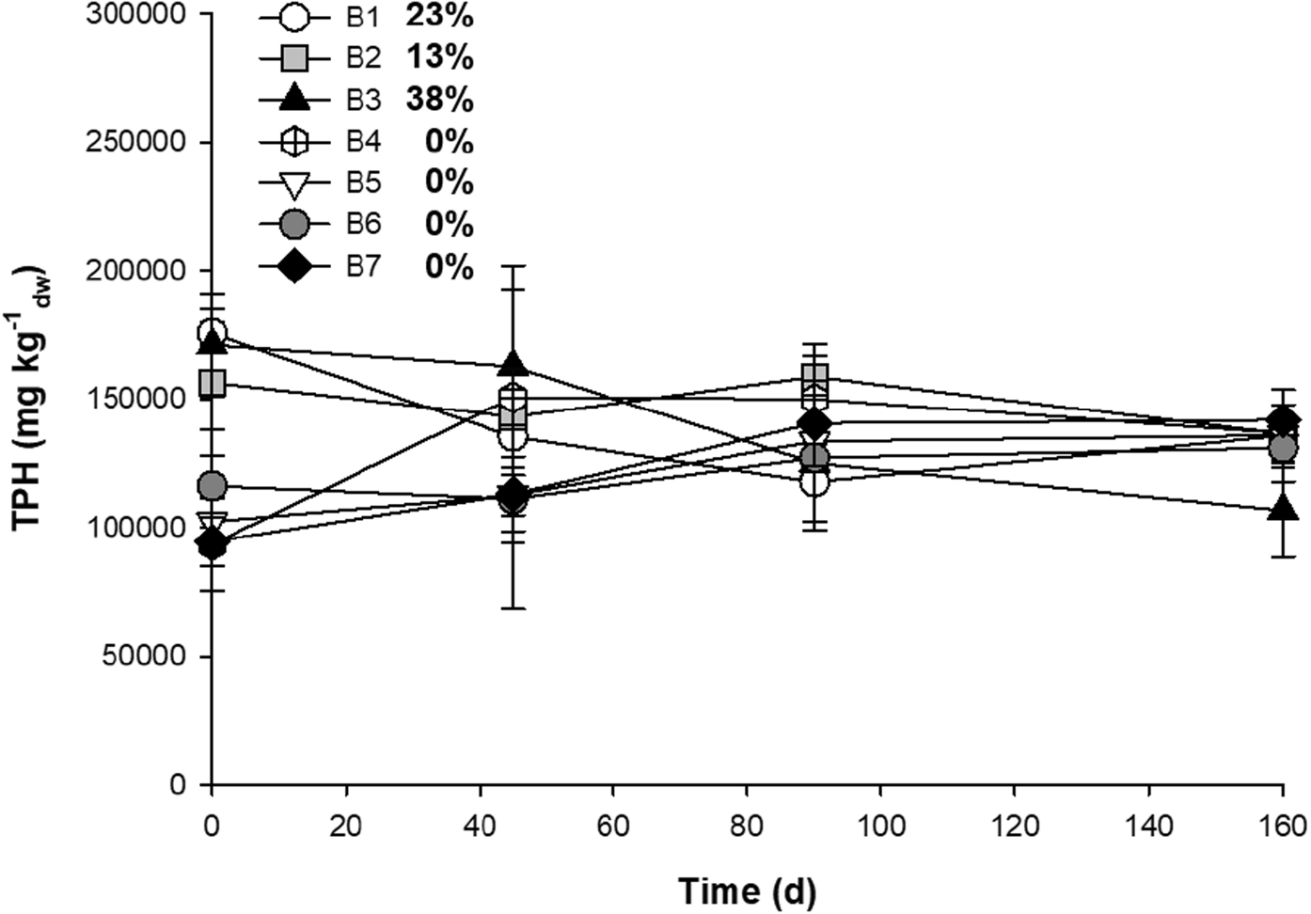
Effect of the addition of different bulking agents and nutrients in heavy oily sludge biodegradation. The degradation of TPH (%) is shown next to each treatment. Error bars represent the standard deviation of three replicates. *B1* soil, organic fertilizer, sawdust, grass, cow manure, biosolids and molasses, *B2* soil, organic fertilizer, sawdust, grass, cow manure, biosolids and molasses, *B3* soil, organic fertilizer, grass, cow manure, raquis, biosolids and molasses, *B4* soil, raquis, rice husks, molasses and inorganic nutrients, *B5* soil, cow manure, rice husks, molasses and inorganic nutrients, *B6* soil, sawdust, cow manure, raquis, rice husks and inorganic nutrients, *B7* soil, organic fertilizer, rice husks and inorganic nutrients

For the second experiment, several bulking agent mixtures and nutrients were evaluated for their ability to enhance the degradation of heavy oily sludge. The initial TPH concentration was highly variable, probably due to the interferences of the organic matter present in some of the bulking agent mixtures during the analytical determination (Fig. 4). In general, a low degradation of heavy oily sludge between 0 and 38% was observed in all bulking agent mixtures. The B3 mixture had the highest degradation of heavy oily sludge (38%) by the end of the study. In contrast, mixtures with a low content of organic matter (B4–B7) did not present any degradation of heavy oily sludge after 160 days of incubation. Because of the low biodegradation obtained, two contrasting treatments (B2 and B5) were selected to continue with the microcosm studies. Mixture B2 had a wide variety of bulking agents, while mixture B5 was composed of only three bulking agents which are ubiquitous and low-cost agricultural by-products found in the vicinity of the treatment plant facility.

**Fig. 5:**
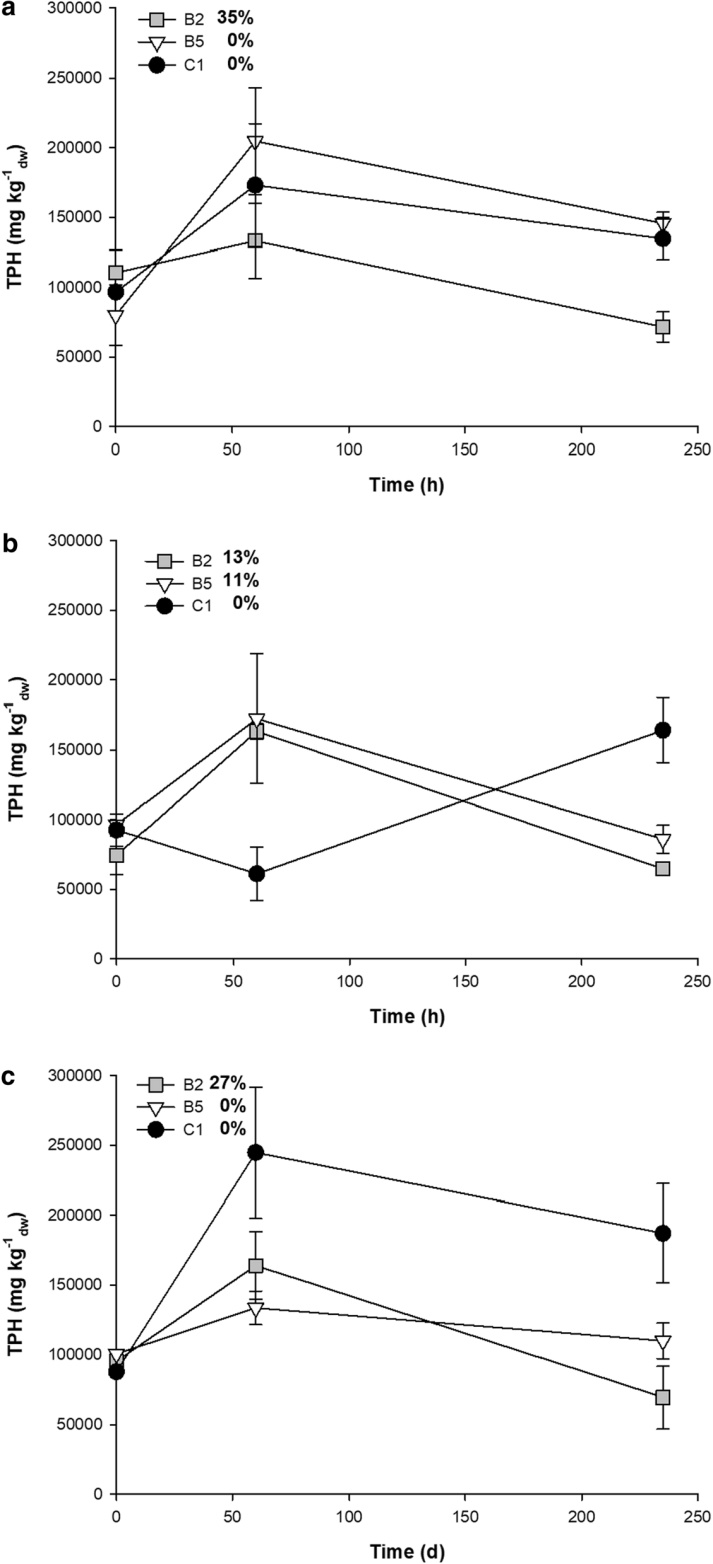
Effect of the addition of bulking agents, nutrients, and surfactants on the heavy oily sludge degradation. **a** Without addition of Tween 80, **b** Tween 80 [0.5%], and **c** Tween 80 [1%]. The degradation of TPH (%) is shown next to each treatment. Error bars represent the standard deviation of three replicates. *B2* soil, organic fertilizer, sawdust, grass, cow manure, biosolids, and molasses, *B5* soil, cow manure, rice husks, molasses, and inorganic nutrients, *C1* soil, sawdust, and inorganic nutrients

To improve the bioavailability of the heavy oily sludge, the effect of the Tween 80 surfactant was evaluated. Biodegradation of the heavy oily sludge was between 0 and 35% in all bulking agent mixtures and with the surfactant concentration evaluated (Fig. 5). The presence and increment of Tween 80 did not enhance heavy oily sludge degradation (Fig. 5). The highest degradation of heavy oily sludge was 35% and achieved in mixture B2 without Tween 80 at 240 days (Fig. 5A). Additionally, mixture B2 showed a 13 and 27% degradation with 0.5 and 1%, respectively, of Tween 80 (0.5 and 1%, respectively) (Fig. 5B–C).

To assess if the low degradation observed in the previous studies due to the toxicity of the heavy oily sludge, we assessed two concentrations of heavy oily sludge, 60,000 and 100,000 mg kg^−1^_dw_, using the bulking agent mixtures B2, B5, and C1. Biodegradation in the lowest concentration of heavy oily sludge (60,000 mg kg^−1^_dw_) varied between 5 and 41% depending on the mixture (Supplementary Fig. S3). The highest degradation of heavy oily sludge was 41% in treatment B2 at 130 days, decreasing from 60,000 to 39,500 mg kg^−1^_dw_ (Supplementary Fig. S3). Despite this, there were no significant differences between the three bulking agent mixtures by the end of study. At 100,000 mg kg^−1^_dw_ biodegradation of the TPH in all treatments ranged from 32 to 37% after 130 days (Supplementary Fig. S3). The greatest degradation of heavy oily sludge was achieved in treatment C1 at 37% although there were no significant differences between the treatments by the end of the experiment (Supplementary Fig. S3).

**Fig. 6:**
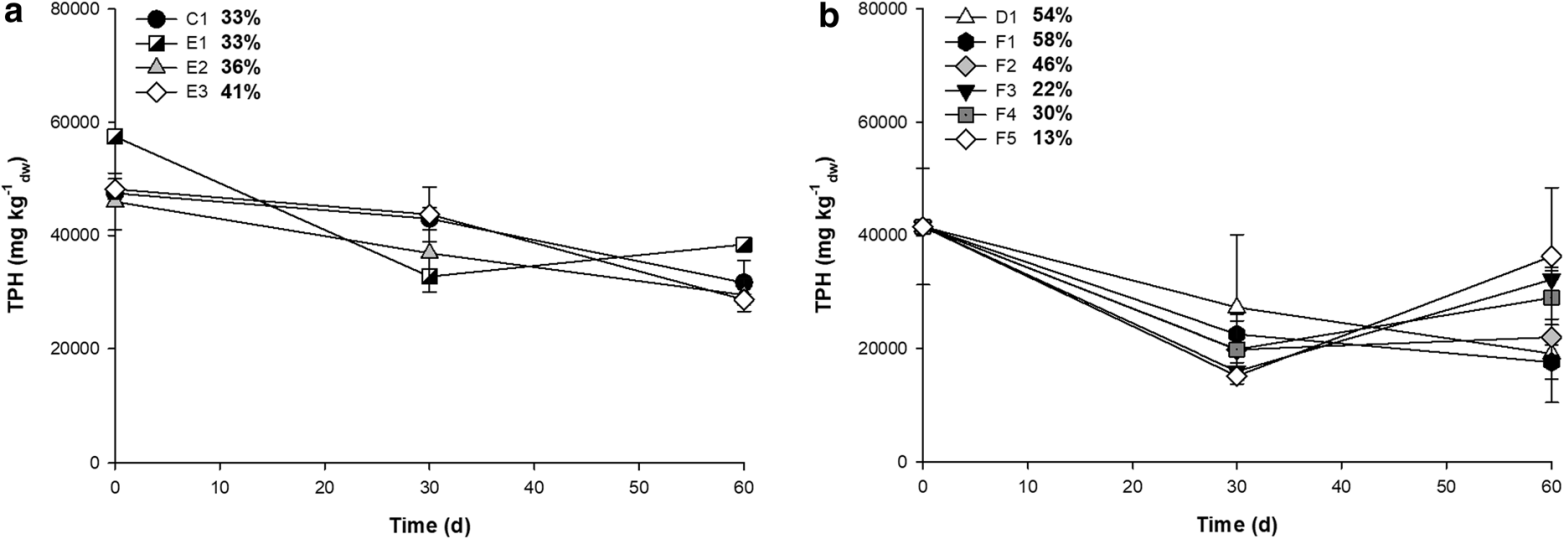
Effect of the bioaugmentation in the heavy oily sludge degradation. **a** Bacterial consortia, and **b** bacteria and fungi consortium. The degradation of TPH (%) is shown next to each treatment. Error bars represent standard deviation of six replicates. *C1* soil, sawdust, and inorganic nutrients, *E1* with enrichment culture, *E2* with bacterial consortium from USBA collection, *E3* with bacterial consortium from enrichment cultures. *D1* soil, rice husks, sugarcane bagasse, and inorganic nutrients, *F1* with enrichment culture, *F2* with bacterial consortium, *F3* with fungal consortium, *F4* mix F1 and *F3*, *F5* mix F2 and F3

Due to the low degradation of heavy oily sludge from the biostimulation studies, a bioaugmentation strategy was tested for 60 days using three bacterial consortia. Biodegradation of heavy oily sludge was achieved and varied between 33 and 41%, depending on the treatment (Fig. 6A). The highest degradation was achieved with bacteria previously isolated and selected from enrichment cultures with heavy oily sludge, with a 41% reduction at 60 days (Fig. 6A). However, the inoculation of the bioaugmentation consortia E1, E2, and E3 did not significantly enhance the degradation of the heavy oily sludge compared with the biostimulation control (Fig. 6A).

Finally, bioaugmentation was evaluated using bacterial and fungal consortia during 60 days. At 40,000 mg TPH kg^−1^_dw_ the biodegradation of heavy oily sludge varied between 13 and 58%, depending on the treatment (Fig. 6B). The greatest degradation of 58% was achieved in treatment F1 at 60 days (Fig. 6B). However, there were no significant differences between the treatment F1 and the corresponding biostimulation control D1, indicating that none of the bioaugmentation treatments improved the degradation of heavy oily sludge.

## Discussion

### Characterization of heavy oily sludge

During this research, 10 heavy oily sludge batches were used for all biodegradation studies. The TPH for those batches were characterized by a gravimetric method and GC-FID analysis, showing a high variation in the TPH concentrations, ranging from 106,600 to 293,700 to 99,600–121,900, respectively (Supplementary Table S1). The TPH range found in the oily sludge during this research was similar to other biodegradation studies (Roldán-Carrillo et al. 2012; Wang et al. 2012; Jasmine and Mukherji 2015; Koolivand et al. 2017; Jerez et al. 2021). This heavy oily sludge used in this study was originated from different steps in the oil treatment process and different oil well facilities. The oils (Castilla, Rubiales, Quifa, Chichimene) from this region are generally characterized with a low API gravity of 12.1, 12.9, 13.9 and 18.4, respectively. In addition, this sludge is a residue of high temperature centrifugation (100–110 °C) in order to recover commercially valuable oil. This characteristic generally will not only affect the biodegradation during the remediation of oily sludge studies, but also the TPH characterization. For this reason, a TPH characterization for each batch was conducted before the bioremediation study.

It is important to mention that the origin of the oil as well as the SARA fractions will determine the nature of the sludge. The resins and asphaltenes were in the high concentration range of 23.7 and 10.2%, respectively. The aromatic fraction of 22.3% was lower than other values found in literature (Tahhan et al. 2011; Roldán-Carrillo et al. 2012; Hu et al. 2013). A high content of resins and asphaltenes in heavy oily sludge makes its degradation by microorganisms more difficult. These HCs fractions are characterized by complex structures with diverse aromatic molecules and heteroatoms of high molecular weight and low solubility (Hernández-López et al. 2015). Due to these characteristics, asphaltenes are considered recalcitrant and their biodegradation has been reported in few studies (Vasco et al. 2011; Jahromi et al. 2014).

Metals (As, Cd, Cr, Hg, Ni, Ag, Pb, and Zn) were found in low concentration and below the limits of Louisiana 29B regulation (Supplementary Table S2) and other studies (Heidarzadeh et al. 2010; Hu et al. 2015; Asgari et al. 2017; Koolivand et al. 2017). According to these results, the presence of metals could not be considered as the limiting factor during the biodegradation of this heavy oily sludge.

### Hydrocarbon degrading microorganisms for microcosm studies

During the study, 72 bacteria and 14 fungi strains able to degrade HCs, were isolated through the enrichment technique with a minimal medium (BH) using heavy oily sludge as the sole source of carbon and energy. This is one of the few studies where fungi were isolated and studied as part of a consortium with bacteria used for their ability to degrade heavy oily sludge. Only ten bacterial strains (Supplementary Table S3) were finally selected based on their growth in the presence of diesel and PAHs, and for their ability to form clear halos in solid media with different PAHs. Most of the isolated bacteria in this study were able to grow in diesel, indicating their ability to degrade the HCs present in this mixture. The high bacterial growth observed in this compound could be attributed to the presence of easily degradable alkanes. Zhang et al. (2010) tested 38 strains that were able to grow on diesel as the sole carbon source and found that 22 of those strains were also able to degrade C12–C25 n-alkanes. Wang et al. (2016b) found that an enhanced landfarming system with sludge and compost amendments effectively removed the low molecular weight TPH (straight-chain aliphatic compounds) present in the diesel, obtaining a high HCs degradation (> 78%) at 175 days.

The selected bacteria showed the highest biodegradation potential included *Pseudomonas* (4), *Stenotrophomonas* (2) and *Sphingobium* (2) (Supplementary Table S3) which have been previously described during the degradation of oily sludge (Zafra et al. 2014; Dörr de Quadros et al. 2016; Wang et al. 2016a; Swathi et al. 2020). Dörr de Quadros et al. (2016) reported that families *Pseudomonadaceae* and *Shingomonadaceae* were abundant in biostimulation treatments where mechanical aeration, pH and humidity were adjusted. Those families are well known for degrading aliphatic and aromatic HCs (Zafra et al. 2014). Cerqueira et al. (2011) isolated a *Stenotrophomonas acidaminiphila* strain capable of degrading saturated and aromatic fractions (92 and 33%, respectively) present in oily sludge. Sarkar et al. (2017) in a similar study found that 46% of the total bacteria isolated belonged to the genus *Pseudomonas*, probably because they can be cultivated under different temperature and pH conditions.

However, these *Pseudomonas* strains were unable to use PAH as a carbon and energy source (Sarkar et al. 2017). These results contrast with the growth of strains 13, 26, and 27 observed on 250 mg L^−1^ of anthracene, and a halo formation by strain 305 in the presence of 250 mg L^−1^ of phenanthrene and pyrene, suggesting PAH degradation. In an investigation by Lafortune et al. (2009), only one bacterial strain was able to degrade phenanthrene and pyrene, while Zhou et al. (2008) observed that the phenanthrene (10/11) was more easily degraded and only a few strains (7/11) formed halos in anthracene. The clear zone observed around the colonies in BH medium with some PAH was a good screening method for selecting the degrader bacteria and their potential ability to degrade the PAH present in the heavy oily sludge. On the other hand, no growth was observed with 30 and 100 mg L^−1^ of toluene, while previous studies have been able to isolate degrader bacteria from contaminated sites capable of using toluene as a sole carbon source (Singh et al. 2018).

Multiple studies have isolated bacteria from oily sludge (Cerqueira et al. 2011; Parhamfar et al. 2020), but little has been reported on fungi recovered from this residue. In this study, 14 fungi with *Aspergillus* sp. and *Paecilomyces* sp. as dominant genera, were isolated from the enrichment cultures with heavy oily sludge, suggesting the important role of fungal strains in the degradation of high molecular weight HCs. The presence of non-specific enzymatic capacity, like laccase, facilitates the breakdown of complex structures and recalcitrant substrates (Vasco et al. 2011). Furthermore, their ability to grow during biostimulation processes and to be part of bioaugmentation with bacteria have been documented, and could contribute to the degradation of TPH and PAHs (Vasco et al. 2011; Zhou et al. 2019).

### Respiration assays during the heavy oily sludge biodegradation

The use of respirometry makes it possible to indirectly monitor the biodegradation of HCs by measuring the consumption of the final electron acceptor and the effect of the contaminant on the microbial metabolism. Respirometry was used to establish the oxygen consumption for different bulking agents and the use of heavy oily sludge during the biodegradation studies. It is important to mention that the presence of heavy oily sludge favored the microbial metabolism as oxygen uptakes increased. The microbial respiration was inhibited by a TPH higher than 100,000 mg kg^−1^_dw_ and for this reason this concentration was used for most of the biodegradation studies. However, in some cases where biodegradation was not observed, a concentration less than 60,000 mg kg^−1^_dw_ was assessed. Most of the bulking agents had a low oxygen consumption; however, the cow manure and compost had a higher oxygen uptake, indicating the presence of readily available carbon and high microbial biomass. A positive effect in the respiration was observed in the contaminated sawdust, rice husks, and rice peat, possibly due to a better pore structure that improved air and nutrient dispersion, and increased the biodegradation of heavy oily sludge by stimulating indigenous microorganisms (Wang et al. 2012). The importance of bulking agents was confirmed by Wang et al. (2012) in field-study biopiles of oily sludge. In addition, it is found in the literature that significant concentrations of HCs present in oily sludge have been degraded in treatments with different bulking agents such as biosolids, compost, and green wastes (Fountoulakis et al. 2009; Ma et al. 2016; Wang et al. 2016a). The low O_2_ uptake observed in the contaminated chicken manure was probably due to interference from the ammonia produced, which can be toxic to microorganisms or cause manometric interferences, as observed by Komilis et al. (2010) in diesel contaminated soils with high concentrations of nitrogen greater than 500 mg kg^−1^.

### Bioremediation studies in microcosms

Biostimulation and bioaugmentation approaches were used to enhance the degradation of heavy oily sludge by using bulking agent that have not been previously applied. In all studies, the TPH concentration showed great variation, possibly due to the nature and heterogeneity of aged heavy oily sludge and the complexity during the mixing process.

The initial bioremediation approaches using bulking agent materials and different TPH concentrations reported in literature showed low TPH biodegradation (Figs. 3 and 4). Despite reducing the initial TPH to 50,000 mg kg^−1^ and incorporating new bulking agent mixtures, the maximum degradation observed after 160 days was still low at 38%. These results contrast with previous studies, where higher biodegradation levels around 70% have been reported by using similar TPH concentrations between 50,000 and 100,000 mg kg^−1^; however, the oily sludge used did not undergo any previous centrifugation processes (Wang et al. 2012; Dörr de Quadros et al. 2016; Asgari et al. 2017). Wang et al. (2012) found that the addition of a bulking agent cotton stalk resulted in the highest TPH removal of 50% for the initial concentration of 58,000 mg kg^−1^ after 220 days. Similarly, Dörr de Quadros et al. (2016) reported the reduction between 30 and 100% of aliphatic and aromatic HCs after 60 days when stimulated with soil from a landfarming site, and nutrients were used to improve the degradation of oily sludge with a concentration of 60,000 mg kg^−1^. Cerqueira et al. (2014), using the same TPH concentration of 60,000 mg kg^−1^ and a similar bulking agent of soil, found that the removal of TPH at 90 days varied from 77 to 79% in the treatments with low and high concentrations of nutrients, respectively. Lastly, Asgari et al. (2017) reported the biostimulation with immature compost removed 72% of the TPH present in the oily sludge at 70 days with an initial concentration of 104,000 mg kg^−1^. Although similar TPH concentrations were evaluated in our study, the heavy oily sludge obtained from oil originating at the sedimentary basin in the oriental plains, with a high density and low API gravity. Additionally, the sludge was from a high temperature centrifugation process that favors the enrichment of resin and asphaltene fractions, making it more recalcitrant to biodegradation (Leon and Kumar 2005; Hernández-López et al. 2015). In other oily sludge biodegradation studies where TPH concentrations were greater than > 170,000 mg kg^−1^, the removal efficiency varied between 58 and 93% after 360 days using bulking agents such as fresh green waste or manure (Liu et al. 2010; Aguelmous et al. 2020). However, it is important to mention that the studies with high degradation were conducted using oily sludge from lighter oil facilities (> API gravity).

The bulking agent mixture D1 (soil, rice husks, sugarcane bagasse, and nutrients) had the highest degradation of 54% of heavy oily sludge at 60 days (Fig. 6B). This was the only mixture which included sugarcane bagasse; this bulking agent has a high content of cellulose, hemicellulose and lignin (Dong et al. 2013). This complex composition may favor the growth of many white-rot fungi and the secretion of enzymes that promote a co-metabolic process. Additionally, the sugarcane bagasse is an important reservoir of native microorganisms and a source of the nutrients N and P that could influence the biodegradation of HCs (Trejo-Hernández et al. 2007). On the other hand, with the bulking agent mixture C1, the degradation of heavy oily sludge was variable. In some experiments, the degradation of TPH was ~ 30% (Figs. 6A and S3), while in other cases there was no biodegradation of heavy oily sludge (Fig. 5). The bulking agent with high organic content mixture B2 had some degradation of TPH at the different concentrations evaluated (Figs. 4 and 5). The high content of organic matter favors the growth of heterotrophic microorganisms, the ratio of nutrients, and the co-metabolism processes of HCs. Therefore, many oily sludge bioremediation studies have evaluated composting as an effective approach to reduce TPH (Asgari et al. 2017; Parhamfar et al. 2020; Poorsoleiman et al. 2020; Aguelmous et al. 2020). To improve the biodegradation of heavy oily sludge, the surfactant Tween 80 was added to the bulking agent mixture. However, no significant difference was observed when compared to B2 mixture without Tween 80 (Fig. 5). These results contrast with previous studies, where significant reduction of TPH between 29 and 62% in contaminated soils had been achieved in a short period of time when surfactants Tween 80, rhamnolipid, Biosolve®, and sodium dodecyl sulfate were added to liquid and microcosm experiments (Gojgic-Cvijovic et al. 2012; De La Cueva et al. 2016; Xu et al. 2018; Rong et al. 2021).

The bioaugmentation with bacteria recovered from oily sludge has shown promising results in the degradation of TPH under controlled conditions (Mishra et al. 2001; Xu et al. 2013; Brigmon et al. 2016; Masy et al. 2016). The addition of an enrichment culture with heavy oily sludge and bulking agent mixture F1 improved biodegradation up to 58% (Fig. 6B). However, these results were not consistently achieved in all studies (Fig. 6), suggesting that bioaugmentation does not always improve degradation efficiency. A study conducted by Koolivand et al. (2020) using compost as the bulking agent and bioaugmentation with a bacterial consortium achieved a reduction of 81% of initial TPH (20,000 mg kg^−1^) at 84 days. In a more recent 84 days study, the bioaugmentation with bacteria increased the degradation of oily sludge (20,000 mg kg^−1^) by 84% compared to biostimulation with finished compost as a bulking agent (36%) (Abtahi et al. 2020). Similar to our findings, Ma et al. (2016) reported a TPH degradation of 56% at 90 days in biopiles with sawdust and inoculation of a bacterial consortium with an initial concentration of 39,600 mg kg^−1^. In contrast to our findings, the bioaugmentation under laboratory conditions has been reported to achieve significantly higher TPH degradation at 75% compared to biostimulation with nutrients at 55% (Roy et al. 2018). However, it is important to mention that only 2 g of oily sludge at 143,800 mg kg^−1^ was evaluated without the addition of any bulking agent during 120 days. Most of the bacteria isolated and used during the bioaugmentation studies have been previously reported as HC degraders. In addition, they were selected based on their ability to degrade diesel and some PAH like anthracene, phenanthrene, naphthalene, and pyrene; however, biodegradation of HCs in the heavy oily sludge was limited under the laboratory conditions.

### Practical implications and limitations of this work

The present study suggests that biological treatment for heavy oily sludge with similar characteristic to the sludge used in the present study is not the best option. This sludge originated from different process of the production of heavy crude oil suffer thermal centrifugation extraction process to recover valuable HC for energy production. The resulting sludge remain with even heavier HC fraction, which are highly recalcitrant. Though these results were quite disappointing, it is valuable to stress out the limitation of biodegradation. In this sense, the main drawback of the present study is the lack of characterization of the HCs the were degraded (~ 40%), as well as the of the molecules that were not. It must be emphasized, however, that this kind of characterization is quit challenging. For example, Sutton et al. (2005) estimated that unresolved complex mixture of HCs isolated from a biodegraded crude oil might contain about 250,000 different compounds. This estimation also stresses out the risk of generalization in regard to the degradation of different type of TPH since it refers to molecules with very diverse range of size and structures (e.g. numbers of carbons, aromatic rings ramifications etc.). As stated above, the characteristic of oily sludge can be highly variable due to multiple factors including the origin of the crude oil (e.g. light vs. heavy), the process in which the sludge is formed (e.g. drilling process or sediment of storage tank), or previous treatment of the sludge such as HCs recovery (e.g. solvent extraction, centrifugation). Therefore, we recommend that future publications about the treatments of oily sludge would describe this type of details that are essential to compare between studies. This information may help to assess to which type of oily sludge a biological treatment might be the best option, and in which cases physicochemical options should be considered.

## Conclusion

Bacteria tested under these laboratory conditions, have limited bioremediation potential for these heavy hydrocarbon sludge materials. The O_2_ consumption observed could be attributed to microbial activity in the different bulking agents tested, which thrived in the presence of a high concentration of 100,000 mg TPH kg^−1^ of heavy oily sludge. Low TPH degradation observed could be due to low microbial viability, the toxic effect of heavy oily sludge, accumulation of the recalcitrant fraction’s resins or asphaltenes, or byproducts and the short time of less than 240 days evaluated. None of the treatments evaluated were able to achieve high (80%) degradation of TPH (mg kg^−1^_dw_) present in the heavy oily sludge, regardless of the addition of bulking agents, microorganisms, or surfactant Tween 80. Results demonstrated that bioremediation (biostimulation and bioaugmentation) of this type of heavy oily sludge, with a pre-treatment such as centrifugation, is not recommended as a treatment option to achieve regulatory levels of 1% w/w.

While bench testing demonstrated the bioremediation capacity of applied microorganisms, the microcosm study confirmed the limitation of this community to make use of amendments for significant hydrocarbon utilization. In future work, a more efficient experimental design and optimization of the oily sludge treatment process is necessary. The rate limiting factors for bioremediation of this heavy oily sludge, e.g., temperature, pH, mixing rate, moisture content, required microbial densities, dissolved oxygen concentrations, contaminant bioavailability, and/or toxicity must be identified.

## Supplementary Information

Below is the link to the electronic supplementary material.

Supplementary material 1 (DOCX 2174 kb)
