# Supplementary material for: Bioremediation of heavy oily sludge: a microcosms study
Source: Biodegradation. 2022 Dec 4;34(1):1–20. doi: 10.1007/s10532-022-10006-1 (PMC9935733; doi:10.1007/s10532-022-10006-1)
Supplement: Supplementary file 1 — Supplementary material 1 (DOCX 2174 kb) [file 10532_2022_10006_MOESM1_ESM.docx]

**Bioremediation of Heavy Oily Sludge: A Microcosms Study**

Cinthya Rondon-Afanador ^a^, Gustavo Pinilla-Meza ^a^, Francy C. Casallas-Cuervo ^a^, Camila Diaz-Vanegas ^a^, Daniela Barreto-Gomez ^a^, Carolina Benavides ^a^, Nicole Buitrago ^a^, Melissa Calvo ^a^, Camila Forero-Forero ^a^, Valentina Galvis-Ibarra ^a^, Victoria Moscoso-Urdaneta ^a^, Maria C. Perdomo-Rengifo ^a^, Laura Torres ^a^, Ziv Arbeli ^a^, Robin L. Brigmon ^b^, Fabio Roldan ^a, *^

^a^ Facultad de Ciencias, Departamento de Biología, Unidad de Saneamiento y Biotecnología Ambiental (USBA), Pontificia Universidad Javeriana, Carrera 7 No. 43-82, Bogotá, DC, Colombia

^b^ Savannah River National Laboratory, Aiken, SC, United States

^*^ [fabio.roldan@javeriana.edu.co](mailto:fabio.roldan@javeriana.edu.co)

**Table S1** TPH concentration of heavy oily sludge batches

| **Batches** | **Gravimetric**  9071B Method | | | **GC-FID**  MADEP Method | | |
| --- | --- | --- | --- | --- | --- | --- |
|  | TPH (mg kg^-1^_dw_) | | CV (%) | TPH (mg kg^-1^_dw_) | | CV (%) |
| 1 | 265,439 | (27,608) | 10.4 | ‒ | ‒ | ‒ |
| 2 | 176,733 | (30,394) | 17.2 | 115,292 | (8,983) | 7.8 |
| 3 | 138,000 | (26,463) | 19.2 | ‒ | ‒ | ‒ |
| 4 | 153,656 | (10,174) | 6.6 | 112,214 | (8,027) | 7.2 |
| 5 | 193,527 | (15,530) | 8.0 | 121,854 | (7,015) | 5.8 |
| 6 | 202,596 | (11,191) | 5.5 | 63,858 | (835) | 1.3 |
| 7 | 279,731 | (35,089) | 12.5 | 86,645 | (12,499) | 14.4 |
| 8 | 204,152 | (21,035) | 10.3 | 73,709 | (17,299) | 23.5 |
| 9 | 131,598 | (10,560) | 8.0 | 113,009 | (11,561) | 10.2 |
| 10 | 106,567 | (8,344) | 7.8 | ‒ | ‒ | ‒ |

**Table S2** Metal concentration of heavy oily sludge

| **Metal** | | **This work** | **Louisiana 29B** |
| --- | --- | --- | --- |
| As | mg kg^-1^ | 0.32 (0.37) | 40 |
| S |  | 5.69 (1.73) | ‒ |
| Cd |  | 0.67 (0.21) | 10 |
| Cr |  | < 20 | 1,000 |
| Hg |  | 0.14 (0.27) | 10 |
| Ni |  | < 40 | 420 |
| Ag |  | < 10 | 200 |
| Pb |  | < 0.2 | 300 |
| Zn |  | 1,086 (246) | 2,300 |

**Table S3** Identification of bacteria selected for bioaugmentation studies

| **Isolate** | **Origin** | **Identification** | **Sequencing 16S rRNA** | **MALDI-TOF mass spectrometry** | **Selection method** | **Hydrocarbons** |
| --- | --- | --- | --- | --- | --- | --- |
| 13 | Heavy oily sludge | *Pseudomonas aeruginosa* |  | X | Higher growth (OD_600nm_) in in BH broth with four HCs | Diesel  Naphthalene  Anthracene |
| 26 |  | *Pseudomonas fluorescens* |  | X |  |  |
| 27 |  | *Pseudomonas fluorescens* |  | X |  |  |
| 96 | USBA collection | *Stenotrophomonas maltophilia* | X |  |  |  |
| 102 |  | *Stenotrophomonas acidaminiphila* | X |  |  |  |
| 107 |  | *Escherichia coli* ATCC 25922* | X |  |  |  |
| 111 |  | *Pseudomonas putida* | X |  |  |  |
| 116 |  | *Favimonas oryzihabitans* | X |  |  |  |
| 155 |  | *Stenotrophomonas maltophilia* | X |  |  |  |
| 305 | Heavy oily sludge | *Pseudomonas citronellolis* |  | X | Formation of clear halos in BH agar with a PAHs | Pyrene  Phenanthrene  Anthracene |
| 309 |  | *Diaphorobacter nitroreducens* | X |  |  |  |
| 201 |  | *Stenotrophomonas maltophilia* |  | X |  |  |
| 203 |  | *Stenotrophomonas maltophilia* |  | X |  |  |
| 400 |  | Unidentified |  | X |  |  |
| 401 |  | *Sphingobium ummariense* | X |  |  |  |
| 407 |  | *Sphingobium ummariense* | X |  |  |  |

*Reference strain of American Type Culture Collection (ATCC).

**Table S4** Different treatments evaluated for each bulking agent during the respirometric degradation assays of heavy oily sludge

| **Bulking agent** | **TPH** | **Inorganic salts^a^** | **Inorganic fertilizer^b^** |
| --- | --- | --- | --- |
| (12 g) | (100,000 mg kg^-1^_dw_) | (C:N:P 100:10:1) | (C:N:P 100:10:10) |
| 1) Sawdust  2) Rice husks  3) Rice peat  4) Chicken manure  5) Cow manure | - | - | - |
|  | + | - | - |
|  | + | - | + |
|  | + | + | - |

^a^ (NH_4_)_2_SO_4_ and K_2_HPO_4_ salts.

^b^ Triple 15 fertilizer (15:15:15; Forza^®^)

**Table S5** Bulking agent mixtures evaluated during the biodegradation of heavy oily sludge

| **Treatments** | **Heavy oily sludge (g)** | **Bulking agents (g)** | | | |
| --- | --- | --- | --- | --- | --- |
|  |  | Soil | Raquis | Rice husk | Pig manure |
| A1 | 0.5 | 0.44 | 4.06 | - | - |
| A2 |  | 0.44 | 2.03 | - | 2.03 |
| A3 |  | 0.44 | - | 4.06 | - |
| A4 |  | 0.44 | - | 2.03 | 2.03 |

5.0 g microcosms (n=3)

**Table S6** Treatments with different mixtures of bulking agents and nutrients

| **Treatments** | **Heavy oily sludge (g)** | **Amendments (g)** | | | | | | | | | | |
| --- | --- | --- | --- | --- | --- | --- | --- | --- | --- | --- | --- | --- |
|  |  | **Soil** | **Organic fertilizer** | **Sawdust** | **Grass** | **Cow Manure** | **Raquis** | **Rice husk** | **Biosolids** | **Molasses** | **(NH_4_)_2_SO_4_** | **K_2_HPO_4_** |
| B1 |  | 0.4 | 0.4 | 0.3 | 0.6 | 0.8 | - | - | 0.8 | 0.05 | - | - |
| B2 |  | 0.4 | 0.4 | 0.2 | 0.3 | 1.0 | - | - | 1.0 | 0.05 | - | - |
| B3 | 1.7 | 0.4 | 0.4 |  | 0.3 | 1.0 | 0.2 | - | 1.0 | 0.05 | - | - |
| B4 |  | 1.3 | - | - | - | - | 1.0 | 0.5 | - | 0.5 | 0.2 | 0.02 |
| B5 |  | 1.3 | - | - | - | 0.5 | - | 0.5 | - | 0.5 | 0.2 | 0.02 |
| B6 |  | 1.3 | - | 0.5 | - | 0.5 | 0.5 | 0.5 | - | - | 0.2 | 0.02 |
| B7 |  | 2.3 | 0.5 | - | - | - | - | 0.5 | - | - | 0.2 | 0.02 |

Molasses and nutrients (C:N:P 100:10:1) were added in solution. 5.0 g microcosms (n=3)

**Table S7** Treatments evaluated with different concentrations of surfactant

| **Treatments** | **Heavy oily sludge (g)** | **Amendments (g)** | | | | | | | | | |
| --- | --- | --- | --- | --- | --- | --- | --- | --- | --- | --- | --- |
|  |  | **Soil** | **Organic fertilizer** | **Sawdust** | **Grass** | **Cow Manure** | **Rice husk** | **Biosolids** | **Molasses** | **NH_4_NO_4_** | **K_2_HPO_4_** |
| B2 |  | 0.33 | 0.33 | 0.25 | 0.49 | 0.85 | - | 0.85 | 0.05 | - | - |
| B5 | 1.9 | 1.6 | - | - | - | 0.85 | 0.65 | - | 0.5 | 0.054 | 0.01 |
| C1 |  | 2.33 | - | 0.77 | - | - | - | - | - | 0.054 | 0.01 |

Molasses and nutrients (C:N:P 100:10:1) were added in solution. 5.0 g microcosms (n=3)

**Table S8** Bioaugmentation treatments with bacterial consortia

| **Bulking agent** | **Heavy oily sludge (g)** | **Treatments** | **Consortia** |
| --- | --- | --- | --- |
| Soil (71 g)  Sawdust (24 g) Inorganic nutrients | 25 | C1 | Without addition of microorganisms |
|  |  | E1 | Enrichment culture |
|  |  | E2 | Bacteria from the USBA collection  (strains: 96, 102, 107, 111, 116) |
|  |  | E3 | Bacteria isolated from enrichment cultures  (strains: 27, 201, 203, 400, 407) |

**Table S9** Bioaugmentation treatments with bacterial and fungal cultures

| **Bulking agent mixture** | **Heavy oily sludge (g)** | **Treatments** | **Consortia** |
| --- | --- | --- | --- |
| Soil (40 g)  Rice husks (20 g) Sugarcane bagasse (20 g)  Inorganic nutrients |  | D1 | Without addition of microorganisms |
|  |  | F1 | Enrichment culture |
|  |  | F2 | Bacterial strains: 96, 111, 116, 27, 305, 309, 401 and 407 |
|  | 40 | F3 | Fungal strains: 36, 39, 54, 100, and 110 |
|  |  | F4 | Mix E2 and E4 |
|  |  | F5 | Mix E3 and E4 |

| 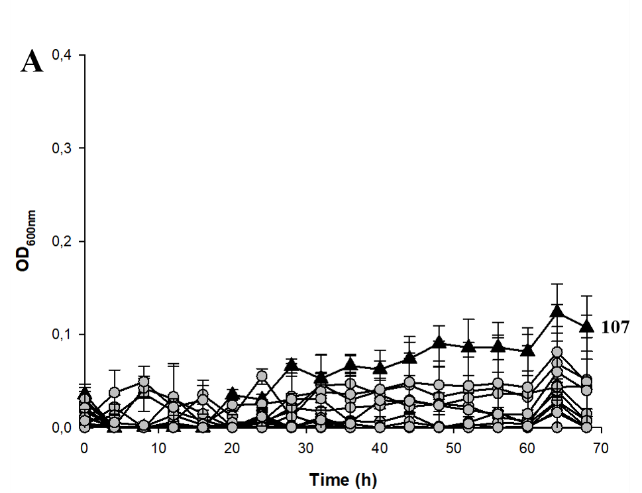 | 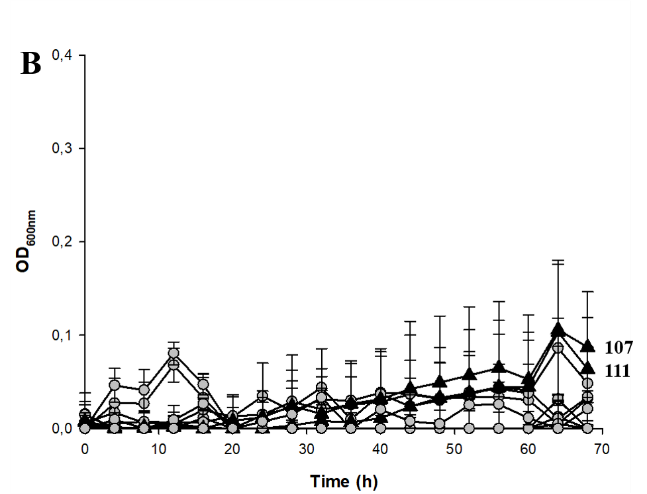 |
| --- | --- |
| 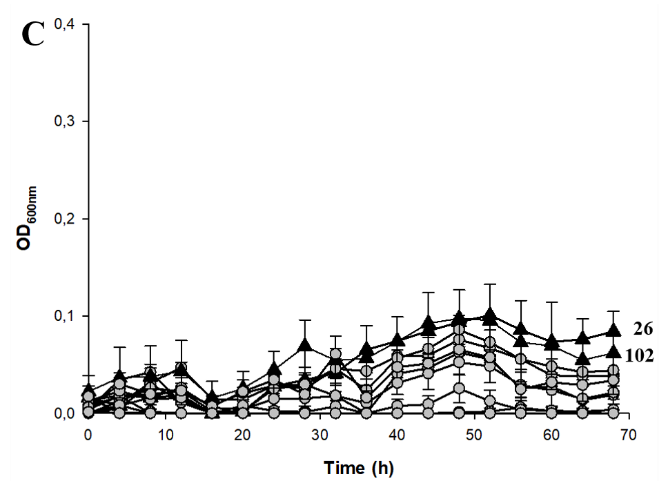 | 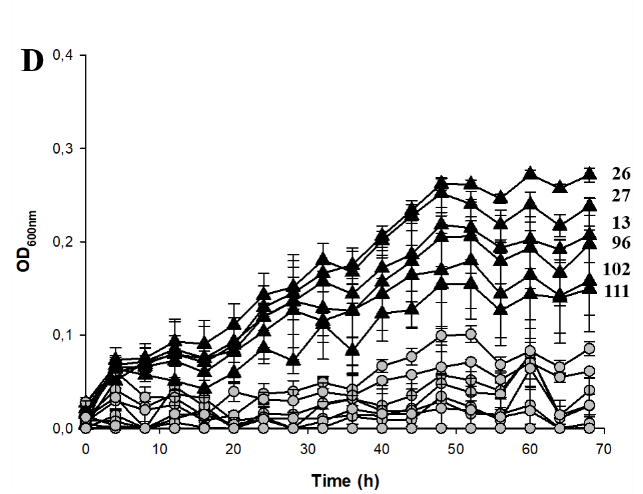 |
| 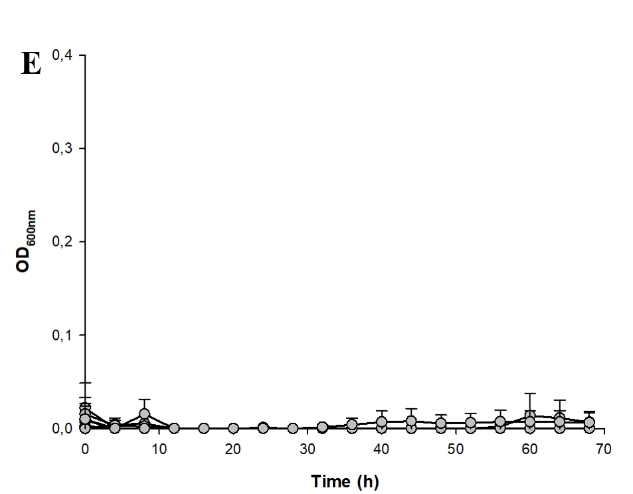 | 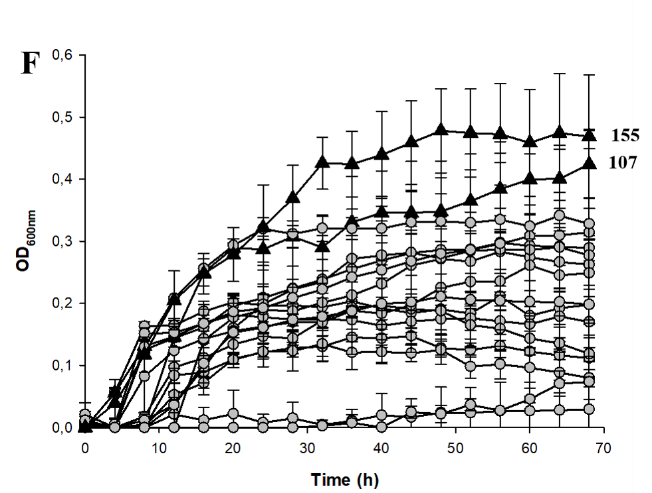 |

**Fig. S1** Bacteria growth on different hydrocarbons as a sole carbon source: naphthalene **a)** 30 mg L^-1^ and **b)** 300 mg L^-1^; anthracene **c)** 50 mg L^-1^ and **d)** 500 mg L^-1^; toluene **e)** 30 mg L^-1^ and diesel **f)** 850 mg L^-1^. Strains with the higher growth are indicated. Error bars represent the standard deviation of six replicates

| 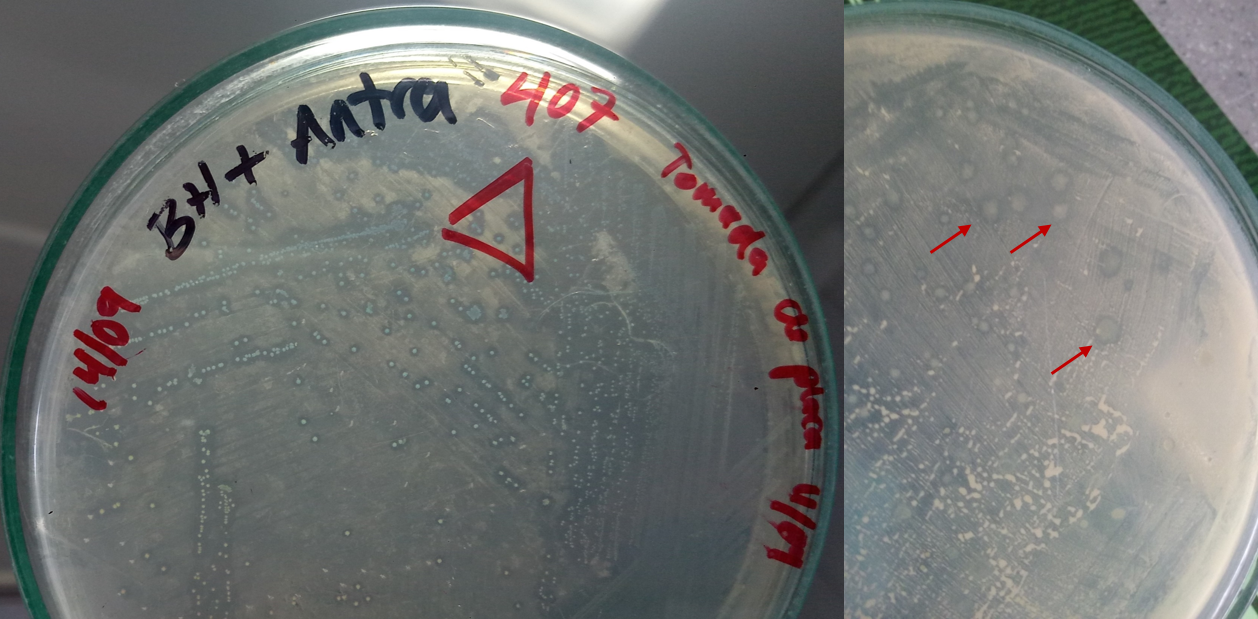 |
| --- |

**Fig. S2** Clear halos around the colonies of PAH degraders

| 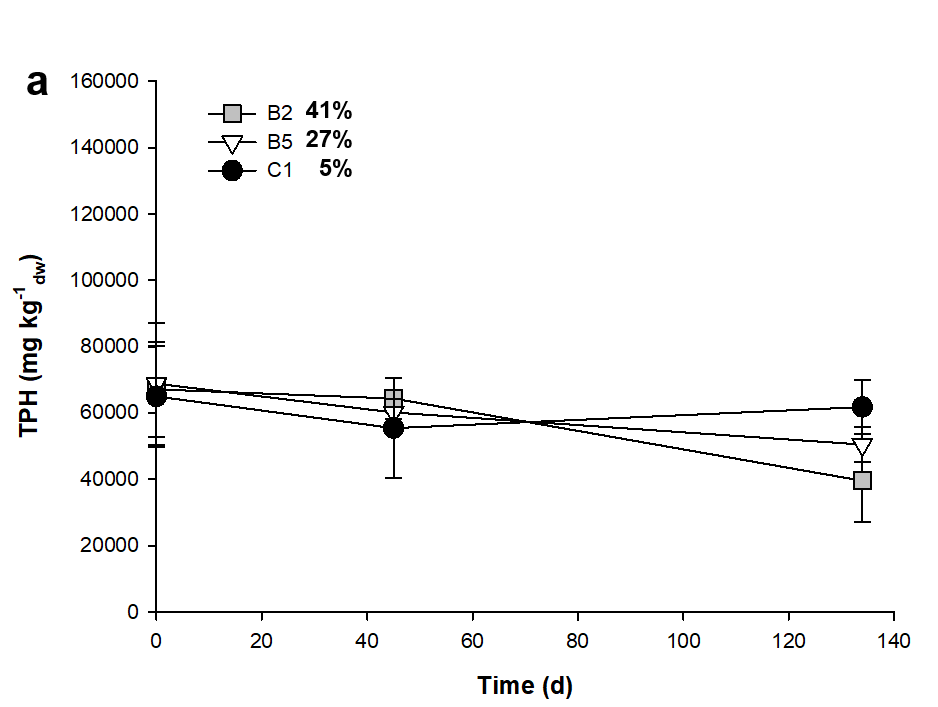 | 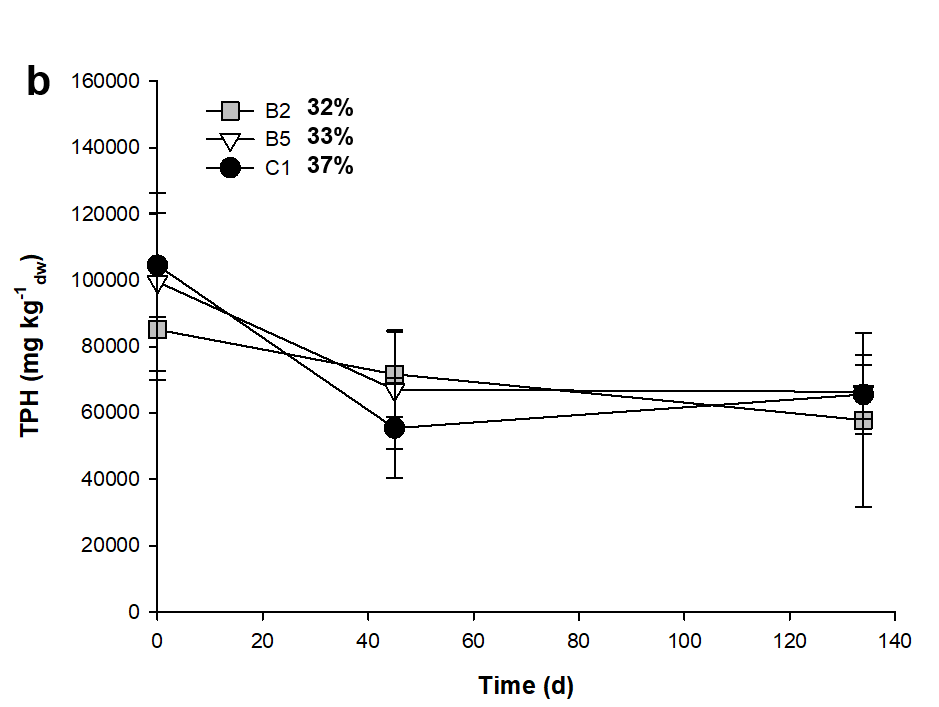 |
| --- | --- |

**Fig. S3** Effect of the heavy oily sludge concentration on microbial degradation. **a)** 60,000 mg TPH kg^-1^_dw_, **b)** 100,000 mg TPH kg^-1^_dw_. The degradation of TPH (%) is shown next to each treatment. Error bars represent the standard deviation of three replicates

B2: soil, organic fertilizer, sawdust, grass, cow manure, biosolids, and molasses; B5: soil, cow manure, rice husks, molasses, and inorganic nutrients; C1: soil, sawdust, and inorganic nutrients
